# Enhancing Quality of Life in Patients with Acute Wounds: The Synergistic Effects of Negative Pressure Wound Therapy and Physiotherapy

**DOI:** 10.3390/biomedicines13040785

**Published:** 2025-03-24

**Authors:** Cristina-Teodora Stanciu, Marcel Mihai Berceanu Vaduva, Daniel Laurentiu Pop, Dinu Vermesan

**Affiliations:** 1 Doctoral School, “Victor Babes” University of Medicine and Pharmacy Timisoara, 300041 Timisoara, Romania; cristina.stanciu@umft.ro; 2 Department XVI—Orthopedics, Traumatology, Urology, and Medical Imaging, Discipline of Orthopedics and Traumatology I, “Victor Babes” University of Medicine and Pharmacy Timisoara, 300041 Timisoara, Romania; daniellaurentiupop@yahoo.com (D.L.P.); dinu@vermesan.ro (D.V.)

**Keywords:** negative pressure wound therapy, rehabilitation, quality of life, edema reduction, functional recovery

## Abstract

**Background and Objectives**: Negative Pressure Wound Therapy (NPWT) is widely used in acute wound management, promoting tissue regeneration and edema reduction. However, the effects of integrating physiotherapy on functional recovery and quality of life remain underexplored. This study assesses the combined impact of NPWT and physiotherapy on functional and clinical outcomes in patients with acute wounds at the Timișoara County Emergency Clinical Hospital. **Methods**: This cross-sectional study included 205 patients divided into two groups: NPWT-only (n = 110) and NPWT plus physiotherapy (n = 95). Clinical and functional parameters, including joint mobility, edema, and pain, were assessed at baseline, ten days, six weeks, and six months. Quality of life and mental health were evaluated using WHOQOL-BREF, SF-36, VAS, and HADS questionnaires. **Results**: Compared to NPWT alone, the NPWT + physiotherapy group showed at discharge greater edema reduction (40.58 ± 2.48 vs. 41.15 ± 2.39), improved joint mobility (14.22 ± 1.66° vs. 10.05 ± 1.76°, *p* < 0.05), and a more significant pain decrease (VAS reduction to 5.68 ± 1.13 vs. 6.7 ± 1.05, *p* < 0.001). Quality of life scores improved notably, with higher WHOQOL-BREF (59.89 ± 5.86 vs. 66.64 ± 6.24, *p* < 0.001) and HADS psychological scores (*p* < 0.001). **Conclusions**: Combining NPWT with physiotherapy enhances functional recovery, reduces pain and anxiety, and improves quality of life. These findings support a multidisciplinary approach in acute wound management.

## 1. Introduction

Acute wounds, whether resulting from trauma or surgical interventions, pose a significant challenge in global medical practice due to their complex healing processes and systemic implications [1]. The healing of these lesions is not merely a biological response but also involves psychosocial factors that affect patients’ overall well-being. Physical impairments following injury can lead to decreased mobility, which, in turn, affects mental health and social participation [2,3,4,5,6]. Chronic pain, functional limitations, and the potential for social isolation further exacerbate this burden, underscoring the need for integrated treatment strategies [3].

Negative Pressure Wound Therapy (NPWT) has become a standard method in acute wound management due to its proven efficacy in reducing edema, promoting tissue regeneration, and lowering the risk of infection. NPWT significantly accelerates the healing process by enhancing granulation tissue formation and reducing bacterial colonization [4,7,8]. Additionally, recent research has shown that NPWT with instillation may further improve outcomes by enhancing tissue hydration and bacterial clearance [1]. Studies have demonstrated that NPWT is particularly effective in complex wounds, facilitating faster closure and minimizing complications such as infections and delayed healing [1,2].

Physiotherapy plays a pivotal role in the functional rehabilitation of patients with acute wounds, contributing to edema reduction, the improvement of joint mobility, and the restoration of physical autonomy. Therapeutic exercises are crucial in restoring function, preventing secondary complications, and enhancing overall recovery in patients with musculoskeletal injuries [2]. The integration of physiotherapy with NPWT has been increasingly recognized as an effective approach to optimizing recovery. Studies have shown that structured rehabilitation programs, including mobilization techniques and muscle strengthening exercises, significantly improve wound healing and functional outcomes in patients undergoing NPWT [7,8]. Techniques such as passive and active mobilizations, targeted exercises, and therapeutic massage can alleviate pain, improve range of motion, and support faster reintegration into daily life activities [2]. Additionally, physiotherapy has been linked to reduced levels of anxiety and depression in post-traumatic patients, further emphasizing the importance of a holistic treatment approach [6].

The primary objective of this study is to evaluate the combined impact of NPWT and classical physiotherapy techniques on the quality of life of patients with acute wounds. This study focuses on measuring physical, psychological, and social dimensions using validated instruments such as the WHOQOL-BREF and SF-36. Moreover, the SF-36 survey offers a comprehensive assessment of both physical and mental health, making it an ideal tool for evaluating the broader effects of medical interventions [5]. By examining both clinical and patient-centered outcomes, this research aims to highlight the importance of integrated therapeutic approaches that address not only tissue healing but also the overall well-being of patients. Furthermore, understanding the psychosocial impact of wounds is crucial in improving long-term rehabilitation outcomes, as patients with severe injuries often experience social stigma and mental distress that can hinder recovery [7].

Research indicates that structured rehabilitation programs can significantly improve long-term functional outcomes and patient satisfaction in wound management [1,2,3,4,6,7,8]. Therefore, this study not only aims to demonstrate the advantages of an integrated NPWT–physiotherapy approach but also to support a multidisciplinary rehabilitation model that enhances functional autonomy, reduces complications, and ultimately improves long-term quality of life in patients with acute wounds.

## 2. Materials and Methods

### 2.1. Study Design and Settings

This cross-sectional study was conducted between September 2020 and November 2024 at the Clinical County Emergency Hospital, Department of Orthopedics-Traumatology I, affiliated with the “Victor Babeș” University of Medicine and Pharmacy in Timișoara. The study period was selected to ensure the inclusion of a representative sample and to facilitate the medium-term monitoring of patients’ quality of life.

The primary objective was to investigate how integrated interventions influence patients’ perception of their overall well-being. Demographic data and quality of life information were collected using a secure electronic database accessible only to authorized medical personnel. Confidentiality was ensured in accordance with the Declaration of Helsinki and the EU GCP Directive 2005/28/EC. The study protocol received approval from the hospital’s ethics committee, and all participants were informed about this study’s purpose and provided written informed consent prior to inclusion.

The study design allowed for a comprehensive evaluation of how interventions affected the physical, psychological, and social dimensions of quality of life in patients with acute wounds. The protocol for Group 2 included classical physiotherapy interventions, consisting of a structured rehabilitation program designed to support physical recovery and enhance patients’ health perceptions. Each session began with manual therapy, followed by a combination of passive and active mobilizations, stretching exercises, and targeted physical exercises aimed at improving joint mobility, reducing stiffness, and restoring functional capacity.

### 2.2. Inclusion and Exclusion Criteria

This study included adult patients aged 19 to 66 with acute wounds localized in the crural region, treated exclusively with Negative Pressure Wound Therapy (NPWT) and free of severe complications such as systemic infections or extensive necrosis. Participation required willingness to undergo the proposed interventions, including functional rehabilitation in Group 2, along with signed informed consent in compliance with ethical regulations.

Exclusion criteria encompassed patients with wounds of different etiologies, such as diabetic ulcers, pressure ulcers, or lesions with extensive necrosis, as well as those with severe comorbidities like cardiovascular diseases or chronic renal failure that could impair healing. Individuals who refused informed consent, failed to adhere to scheduled evaluations, or developed major complications during this study, such as severe infections or the need for amputation, were also excluded. Additionally, patients in Group 2 who did not participate in the functional rehabilitation program were not eligible for inclusion.

All patients included in this study were documented according to international clinical standards, using the ICD-10 system for injury classification [9]. The criteria were carefully selected to ensure the homogeneity of the sample and to minimize data variability, thus providing a robust framework for the comparative evaluation of interventions. Patient confidentiality was guaranteed by adhering to both national and international regulations, including the EU GCP Directive 2005/28/EC and the Declaration of Helsinki.

### 2.3. Data Collection and Surveys

To assess the combined impact of Negative Pressure Wound Therapy (NPWT) and functional rehabilitation on the quality of life and functional parameters of patients with acute wounds, this study included the rigorous collection of clinical and functional data. Demographic information, such as age, gender, body mass index (BMI), lifestyle habits (smoking, alcohol consumption), marital status, and residence (urban or rural), was extracted from electronic medical records and supplemented by direct evaluations during therapeutic interventions. Data confidentiality was ensured through the implementation of international data protection standards in accordance with EU GCP Directive 2005/28/EC.

Clinical and functional measurements were systematically performed to document patients’ progress following interventions. In this study, the measurement of calf and edema circumferences, as well as edema depth, was carried out using standardized techniques to ensure accuracy and reproducibility across assessments. Calf circumference was measured at the widest part of the calf using a flexible measuring tape, while edema circumference was determined at the point of maximum swelling. Both measurements were taken with the patient in a seated position, legs uncrossed, and feet flat on the floor. Edema depth was assessed using a depth gauge; the instrument was gently pressed against the swollen area until resistance was felt, and the depth from the surface of the skin to the firm resistance was recorded. These measurements were taken at predetermined intervals of 10 days, 42 days, and 180 days post-treatment, providing data on the progression and resolution of edema over time. The range of motion (ROM) was evaluated using a standardized goniometer applied to the ankle and knee joints, while muscle strength was assessed using standardized Manual Muscle Testing (MMT) protocols [10].

To analyze the physical, mental, and social dimensions of quality of life, internationally validated tools were employed. The WHOQOL-BREF provided an extensive overview of general health and quality of life, covering domains such as physical well-being, psychological health, social relationships, and environment [11]. Additionally, the SF-36 Health Survey evaluated physical and emotional aspects of health and overall well-being, offering a comprehensive measure for assessing both physical functioning and mental health across diverse populations [5].

The questionnaires were administered at four critical time points: baseline, day 10, 42 days, and 180 days. This allowed for a detailed analysis of their functional progress and health perceptions [12]. To evaluate pain intensity, the Visual Analog Scale (VAS) was used. This internationally validated tool enables patients to quantify their pain on a scale from 0 to 10, where 0 indicates no pain, and 10 represents the most severe pain imaginable [13]. The results were documented and correlated with other clinical and functional parameters. Mental health was also investigated using the Hospital Anxiety and Depression Scale (HADS), which measures levels of anxiety and depression in a clinical context.

All evaluations were conducted by qualified personnel using validated equipment. The resulting data were meticulously documented in a secure electronic database, adhering to ethical guidelines and ensuring the integrity of the collected information.

### 2.4. Statistical Analysis

The management and analysis of the data collected in this study were performed using IBM SPSS statistical software, version 26.0 (IBM Corp., Armonk, NY, USA) [14]. The sample size was determined based on feasibility and the established inclusion criteria, with a total of 205 participants completing all stages of this study. The obtained data were expressed as mean ± standard deviation (SD) for continuous variables, while categorical variables were presented as frequencies and percentages to ensure a detailed description of the sample [15]. To analyze differences between the two groups, appropriate statistical methods were applied for each type of variable. Differences in means for continuous variables, such as range of motion and edema circumference, were analyzed using Student’s *t*-test. For continuous variables with non-normal distributions, adjusted parametric tests were applied. The comparison of categorical variables, such as the distribution of wound types between groups, was conducted using the chi-square test to evaluate associations between groups.

For longitudinal measurements, such as changes in joint range of motion and edema reduction over time, Repeated Measures ANOVA was used. This allowed for a detailed evaluation of progress throughout the study period. Relationships between objective variables (e.g., range of motion and edema reduction) and subjective measures (e.g., questionnaire scores from WHOQOL-BREF, SF-36, and the HADS [16]) were investigated using Pearson and Spearman correlation coefficients, depending on the data distribution. A statistical significance threshold of *p* < 0.05 was used for all analyses. To reduce the risk of Type I errors due to multiple testing, the Bonferroni correction was applied.

## 3. Results

### 3.1. Patient Demographics

A total of 216 patients were included in this study according to the established inclusion and exclusion criteria. No patients were excluded due to incomplete medical records; however, 11 were lost to follow-up, resulting in 205 patients eligible for analysis. Group 1 included 110 patients who received NPWT exclusively, while Group 2 consisted of 95 patients who received NPWT combined with classical physiotherapy techniques. The groups were balanced in terms of the number of subjects and general clinical characteristics. The mean age of patients in Group 1 was 36.59 ± 5.2 years, while, in Group 2, it was 35.8 ± 3.7 years, with no statistically significant differences between groups (*p* > 0.05).

Regarding the type of wounds, the distribution of different trauma categories did not significantly differ between the two groups (*p* = 0.933). Specifically, in Group 1, 37.3% of patients had open tibial fractures, 42.7% had post-surgical wounds (osteosynthesis, external fixations), and 20% suffered from crush injuries. In Group 2, the distribution was 40% for open tibial fractures, 41.1% for post-surgical wounds, and 18.9% for crush injuries (Table 1).

### 3.2. Functional and Clinical Parameters

**Figure 1:**
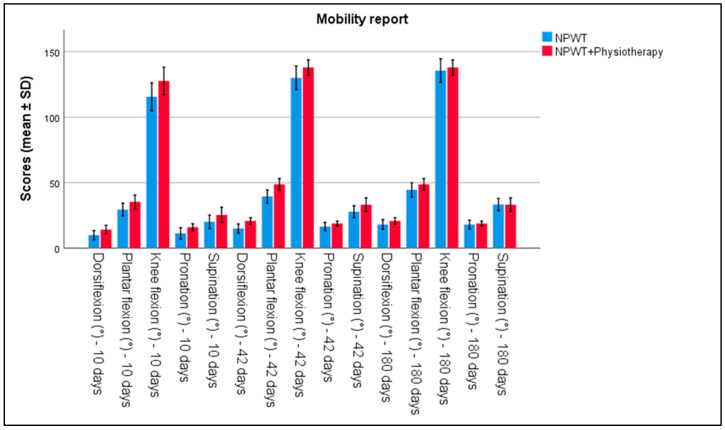
Analysis of the mobility report over the 3 periods of time. Error bars represent standard deviation.

The evaluation of functional parameters showed clear differences between the two groups. Joint mobility, measured using a goniometer, demonstrated significant improvements. In Group 2, the ankle range of motion was higher in all measurements (dorsiflexion, plantar flexion, pronation, and supination) by day 10, compared to Group 1 (*p* < 0.001). Similarly, knee mobility was also higher in Group 2, compared to Group 1, as seen in Table 2 and Figure 1.

Table 3 of this study provides detailed measurements of calf circumference, edema circumference, and edema depth at 10 days, 42 days, and 180 days for patients treated with either NPWT alone (n = 110) or NPWT combined with physiotherapy (n = 95). At 10 days, the calf circumference was 37.48 cm (SD = 1.84) for the NPWT group and 37.25 cm (SD = 1.76) for the NPWT plus physiotherapy group. The edema circumference was 41.15 cm (SD = 2.39) and 40.58 cm (SD = 2.48), respectively. Edema depth was 1.49 mm (SD = 0.8) and 1.41 mm (SD = 0.59). At 42 days, the calf circumference was slightly reduced to 36.28 cm (SD = 1.8) for NPWT alone and 36.39 cm (SD = 1.65) with physiotherapy, with corresponding edema circumferences of 37.49 cm (SD = 1.84) and 37.25 cm (SD = 1.76). The edema depth also showed a reduction to 0.9 mm (SD = 0.042) and 0.63 mm (SD = 0.28). By 180 days, calf circumferences further decreased to 35.23 cm (SD = 1.79) for NPWT and 36.07 cm (SD = 1.62) for combined treatment, with edema circumferences at 36.29 cm (SD = 1.8) and 36.40 cm (SD = 1.65). The depth of edema was significantly reduced to 0.53 mm (SD = 0.24) in the NPWT group and 0.16 mm (SD = 0.12) in the combined treatment group S, as seen in Table 3.

**Figure 2:**
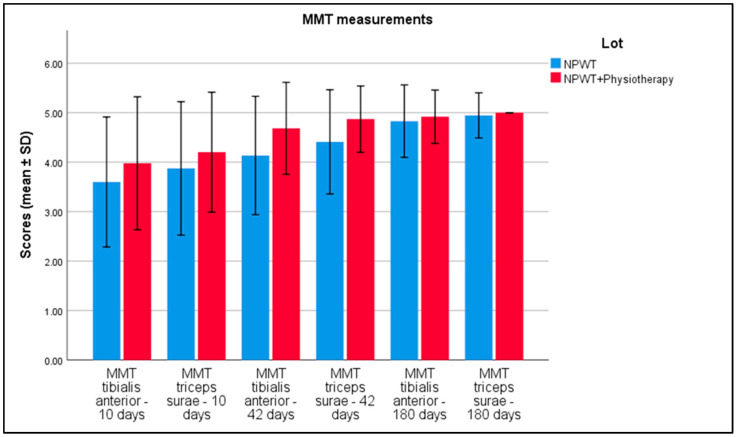
Analysis of the MMT results over the 3 periods of time. Error bars represent standard deviation.

Table 4 displays Manual Muscle Testing (MMT) scores for the tibialis anterior and triceps surae muscles at intervals of 10 days, 42 days, and 180 days in patients treated with Negative Pressure Wound Therapy (NPWT) alone (n = 110) and those receiving NPWT combined with physiotherapy (n = 95). Initial MMT scores for tibialis anterior were 3.27 (NPWT alone) and 3.09 (NPWT plus physiotherapy), with a *p*-value of 0.051. Initial scores for triceps surae were 3.46 and 3.31, respectively, with a *p*-value of 0.103. At 10 days, MMT scores were 3.60 for tibialis anterior and 3.88 for triceps surae in the NPWT group, and 3.98 and 4.20 in the combined treatment group, both with *p*-values < 0.001. By 42 days, scores increased to 4.14 and 4.41 for NPWT alone and 4.68 and 4.87 for combined treatments, with *p*-values < 0.001. At 180 days, scores were 4.83 and 4.95 for NPWT alone and 4.92 and 5.00 for the combined group, with *p*-values of 0.052 and 0.021, respectively (Figure 2).

### 3.3. Questionnaire Analysis

**Figure 3:**
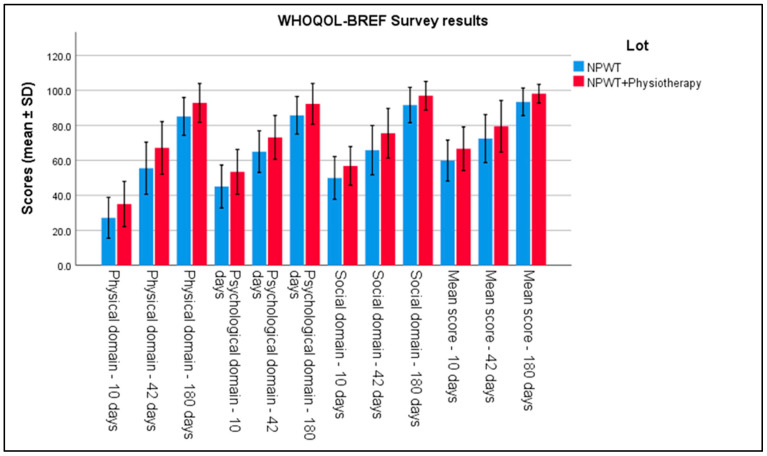
Analysis of the WHOQOL-BREF survey results over the 3 periods of time. Error bars represent standard deviation.

In the analysis of questionnaires evaluating quality of life, the physical component scores from the WHOQOL-BREF indicated significant improvements in Group 2 at 6 weeks (42 days) post-intervention. Differences between groups were evident, with a mean score of 35.04 ± 6.46 in Group 2 compared to 27.17 ± 5.83 in Group 1 (*p* < 0.001). This result reflects more advanced functional recovery in the group that received physiotherapy combined with negative pressure therapy. Regarding the social domain of the WHOQOL-BREF, Group 2 showed significantly better scores compared to Group 1 at 42 days, highlighting the favorable impact of physiotherapy on social interaction and quality of life (*p* < 0.001). The evaluations of the mental component also showed notable improvements in Group 2, with an average mental component score 18% higher than in Group 1 (*p* < 0.001), suggesting that active involvement in physiotherapy had a beneficial effect on patients’ psychological and emotional well-being. All these can be seen in Table 5 and Figure 3.

**Figure 4:**
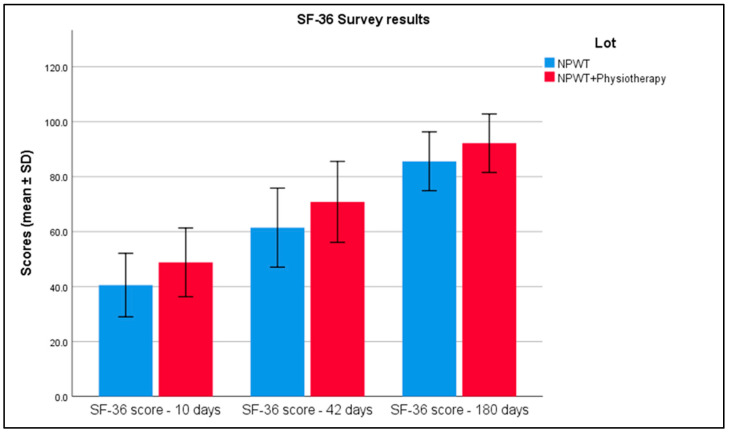
Analysis of the SF-36 survey results over the 3 periods of time. Error bars represent standard deviation.

At 10 days, the average SF-36 score was 40.54 with a standard deviation of 5.78 in the NPWT group, compared to 48.8 with a standard deviation of 6.26 in the combined therapy group. By 42 days, scores increased to 61.44 (SD = 7.2) for NPWT alone and to 70.82 (SD = 7.36) for the combined treatment. By 180 days, scores further improved to 85.60 (SD = 5.35) in the NPWT group and to 92.18 (SD = 5.32) in the NPWT plus physiotherapy group, with statistically significant differences observed at all time points (Table 6 and Figure 4).

**Figure 5:**
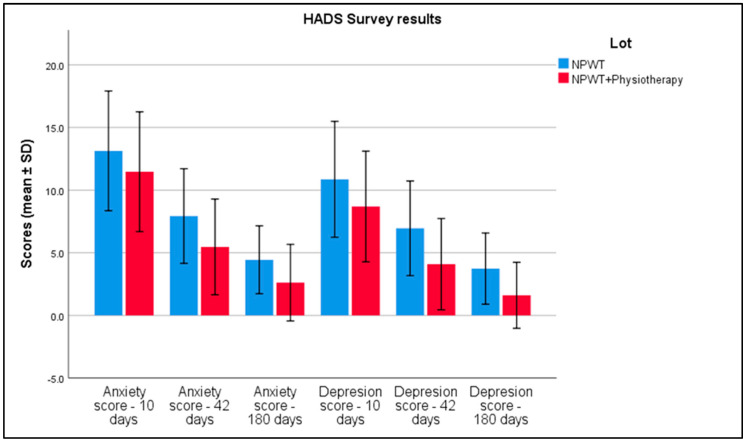
Analysis of the HADS survey results over the 3 periods of time. Error bars represent standard deviation.

For anxiety scores at 10 days, the NPWT-only group reported an average score of 13.14 with a standard deviation of 2.39, compared to 11.47 (SD = 2.39) in the NPWT plus physiotherapy group. By 42 days, these scores had decreased to 7.93 (SD = 1.89) in the NPWT group and 5.47 (SD = 1.91) in the combined group. At 180 days, further reductions were observed, with scores dropping to 4.44 (SD = 1.35) and 2.62 (SD = 1.53), respectively. Depression scores followed a similar trend. At 10 days, the NPWT group had an average depression score of 10.87 (SD = 2.31), which was higher than the 8.69 (SD = 2.21) observed in the combined treatment group. By 42 days, scores reduced to 6.95 (SD = 1.89) in the NPWT group and 4.09 (SD = 1.82) in the combined group. At 180 days, scores further decreased to 3.74 (SD = 1.42) for NPWT alone and 1.61 (SD = 1.32) for the combined treatment, with statistically significant differences among all comparison groups, as seen in Table 7 and Figure 5.

The Visual Analog Scale (VAS), used to measure pain, indicated a greater reduction in pain in Group 2, with an average decrease by day 10, compared to Group 1 (*p* < 0.001), as seen in Table 8. At 10 days post-treatment, the average VAS score was 6.7 with a standard deviation of 1.05 in the NPWT group and 5.68 with a standard deviation of 1.13 in the combined therapy group. By 42 days, the VAS scores had decreased to 3.31 (SD = 1.34) in the NPWT-only group and 1.74 (SD = 1.25) in the NPWT plus physiotherapy group. At 180 days, the scores further reduced to 0.05 (SD = 0.31) in the NPWT group, while the combined therapy group reported a score of 0.

At 10 days post-intervention, dorsiflexion showed a moderate positive correlation with SF-36 scores (r = 0.444, *p* < 0.001) and WHOQOL-BREF scores (r = 0.352, *p* < 0.001), and a negative correlation with HADS anxiety scores (r = −0.279, *p* < 0.001) and VAS pain scores (r = −0.319, *p* < 0.001). Similarly, plantar flexion and knee flexion at 10 days also showed significant correlations with the same health outcomes. By 42 days, the correlation coefficients generally increased, indicating stronger relationships between the physical functions and health outcomes. For instance, dorsiflexion at this time point had an increased positive correlation with SF-36 scores (r = 0.464, *p* < 0.001) and a stronger negative correlation with VAS pain scores (r = −0.457, *p* < 0.001). At 180 days, the correlations varied, with dorsiflexion still showing a positive correlation with SF-36 scores (r = 0.373, *p* < 0.001) and WHOQOL-BREF scores (r = 0.423, *p* < 0.001), but a weaker negative correlation with VAS pain scores (r = −0.124, *p* < 0.001). Notably, the correlation between plantar flexion and VAS pain scores at 180 days was not statistically significant (r = −0.023, *p* = 0.743), highlighting variable long-term impacts on pain perception (Table 9).

## 4. Discussion

Our study marks a significant advancement in the field of acute wound management by exploring the synergistic effects of combining NPWT with physiotherapy—a novel approach not extensively examined in prior research. While the existing literature extensively documents the benefits of NPWT alone in enhancing tissue regeneration and reducing edema, our findings reveal that integrating physiotherapy can further amplify these benefits, leading to notable improvements in joint mobility, pain reduction, and edema management [17,18,19]. Moreover, our results demonstrate a statistically significant enhancement in patients’ quality of life, as measured by the WHOQOL-BREF, SF-36, VAS, and HADS scores, suggesting that the combined treatment approach not only accelerates physical recovery but also contributes profoundly to the psychological well-being of patients [20]. This holistic improvement underscores the potential of a multidisciplinary treatment regimen in delivering superior outcomes in acute wound care, thereby setting our study apart from conventional NPWT studies that do not incorporate physiotherapy [21].

The significance of evaluating clinical outcomes at multiple time points in our study cannot be overstated, as it allows for a comprehensive understanding of the recovery trajectory and the sustained effects of combining Negative Pressure Wound Therapy (NPWT) with physiotherapy [22,23,24,25,26]. By systematically assessing parameters such as pain, anxiety, and depression at intervals of 10 days, 42 days, and 180 days post-intervention, we are able to capture not only the immediate benefits of this integrative treatment approach but also its long-term impacts [27,28,29,30]. This temporal perspective is crucial in demonstrating the progressive improvements and enduring advantages of a multidisciplinary approach to acute wound management, providing valuable insights into both the short-term alleviation and the lasting enhancement of patient health outcomes [31,32,33].

In Group 2, where NPWT was combined with physiotherapy, we observed significant improvements in joint mobility, muscle strength, and social interaction. Zhang et al. reported that recent advancements in NPWT, particularly with instillation, have enhanced its ability to reduce bacterial burden, modulate inflammation, and optimize wound healing [34]. This aligns with our findings, as physiotherapy combined with NPWT resulted in superior functional improvements and faster recovery. Additionally, proprioceptive neuromuscular facilitation techniques significantly enhance post-trauma functional recovery, particularly in improving joint flexibility and muscle coordination [19]. Furthermore, Lalezari et al. confirmed that NPWT with instillation promotes wound bed preparation more effectively than standard NPWT alone, thereby accelerating recovery and functional outcomes [35].

The impact on quality of life was evident through increased scores in the WHOQOL-BREF and SF-36 questionnaires. In Group 2, the physical component of WHOQOL-BREF improved faster compared to Group 1, reflecting enhanced functional recovery and reintegration into daily activities. This aligns with the findings of Bergquist-Beringer et al., who reported that wound care interventions, particularly when combined with rehabilitation, have a profound impact on health-related quality of life in trauma patients [21]. Furthermore, Santema et al. emphasized that NPWT significantly enhances wound closure rates and quality of life compared to conventional treatment methods [36]. The mental health improvements in Group 2 are consistent with Kuwahara et al., who observed that advanced physiotherapy techniques correlate strongly with reduced anxiety and depression levels, contributing to a holistic recovery process [22].

From a clinical perspective, the observed reduction in edema and the increase in joint mobility in Group 2 underscore the essential role of active and passive mobilizations in promoting local circulation and lymphatic drainage. As Orgill and Bayer suggested, the mechanical forces applied through NPWT and physiotherapy stimulate angiogenesis and tissue remodeling, thereby enhancing functional recovery [1]. Additionally, Krug et al. confirmed that combining NPWT with physical therapy leads to improved joint function and reduced inflammation in trauma patients [18]. Supporting this, Dumville et al. noted that NPWT not only accelerates wound closure but also reduces edema and enhances overall tissue perfusion, leading to improved patient mobility and function [37].

Pain reduction, assessed via the Visual Analog Scale (VAS), showed a more substantial decline in Group 2. Hawker et al. noted that integrated pain management strategies, including physiotherapy, are critical in facilitating functional recovery and reducing chronic pain development [13]. This supports our findings that patients receiving combined interventions reported lower pain levels, contributing to faster and more comfortable recovery trajectories. Furthermore, another study emphasized that NPWT with instillation decreases bacterial colonization and inflammation, which significantly reduces pain levels and improves patient outcomes [35].

In terms of mental health, Hospital Anxiety and Depression Scale (HADS) scores indicated a significant reduction in anxiety and depression in Group 2. One study found that structured rehabilitation programs can alleviate psychological distress by fostering a sense of control and progress in patients [14]. This suggests that physiotherapy not only addresses physical recovery but also plays a vital role in emotional healing. Moreover, Santema et al. reported that patients treated with NPWT exhibit greater psychological well-being and faster recovery rates than those receiving standard wound care [36].

Moreover, the direct interaction between the physiotherapist and patient during rehabilitation sessions was instrumental in building patient confidence and engagement. Peters et al. found that patient involvement and positive therapeutic relationships are key predictors of both physical and emotional recovery following surgical interventions [20]. Furthermore, Zhang et al. emphasized that the incorporation of rehabilitation techniques into NPWT protocols enhances patient engagement, reduces complications, and improves long-term recovery outcomes [34].

Overall, our findings corroborate the synergistic effects of combining NPWT with physiotherapy. This integrated approach not only accelerates functional recovery but also enhances emotional well-being and quality of life, consistent with the literature that advocates for comprehensive treatment models in trauma care [21,22]. Additionally, Dumville et al. concluded that NPWT plays a key role in reducing wound-related complications, supporting tissue regeneration, and improving functional and psychological outcomes in patients recovering from acute injuries [37].

Conversely, anxiety scores from the Hospital Anxiety and Depression Scale (HADS) showed a significant negative correlation with ankle mobility (r = −0.45, *p* < 0.01), suggesting that physical limitations may contribute to poorer psychological states. These findings confirm the existing literature that underscores the complex interaction between physical and mental health. Zigmond and Snaith observed that physical impairments often lead to heightened anxiety and depression, creating a cyclical impact on recovery [14], while de Rezende Barbosa et al. further emphasized that functional limitations in lower limb rehabilitation are closely linked to elevated anxiety levels [23].

Our findings underscore the necessity of integrated treatment strategies that address both the physical and psychosocial aspects of recovery. The significant differences between the two groups, particularly in short-term evaluations, highlight the benefits of physiotherapy in accelerating recovery and improving quality of life. Monitoring at 6 months demonstrated the persistence of these benefits, suggesting that active interventions contribute to the medium-term stabilization of both functional and psychological progress. 

From a clinical perspective, integrating standardized questionnaires such as the WHOQOL-BREF, SF-36, and HADS into routine practice can offer valuable insights into the broader impact of treatment on patients’ overall health. These tools enable clinicians to personalize therapeutic approaches, adapting interventions to the individual needs of patients. Ware and Sherbourne emphasized that the SF-36 allows for the detailed assessment of both physical and emotional health, guiding clinicians in tailoring interventions effectively [5], while Zigmond and Snaith reiterated that the HADS is instrumental in identifying underlying psychological challenges that may hinder recovery [14].

Furthermore, a systematic review concluded that NPWT has major effects on the physical, psychological, and social domains of QoL. Knowledge of these effects may lead to improved treatment decisions for patients with hard-to-heal wounds regarding the use of NPWT or standard wound care [36]. This highlights the importance of considering the comprehensive impact of NPWT on patients’ lives when planning treatment strategies. Additionally, it has been observed that negative psychological states can impair immune function and wound healing [35]. This underscores the importance of addressing psychological well-being as part of a holistic approach to wound care.

Our study supports the integration of physical and psychosocial interventions in wound care to enhance both functional recovery and quality of life. Utilizing standardized assessment tools can aid in tailoring treatments to individual patient needs, ultimately leading to more effective and comprehensive care. While the results of this study provide valuable insights into the benefits of combining NPWT with physiotherapy in the treatment of acute wounds, several important limitations must be acknowledged.

The relatively small sample size represents a significant constraint that may reduce the generalizability of the conclusions and the ability to detect subtle effects or smaller differences between groups. Future studies with larger participant numbers could confirm and expand upon these findings. Furthermore, the absence of a control group without intervention limits the interpretation of results. Comparing the two groups with a cohort of patients receiving no active treatment would have provided a broader context for evaluating the effects of NPWT and physiotherapy. The heterogeneity in the application of physiotherapy interventions is another factor to consider. While protocols were standardized, adherence levels and the accuracy of physiotherapy techniques may have varied between patients, introducing a source of variability. More rigorous monitoring and the use of digital tracking methods could improve the uniformity of intervention delivery. Finally, the specificity of the studied population, focused on acute wounds localized to the crural region, limits the generalizability of results to other types of wounds, such as diabetic ulcers or chronic wounds. Future research should extend this approach to other patient categories to confirm the applicability of these interventions The influence of external factors such as the level of physical activity, nutrition, and comorbidities, as well as internal factors like wound localization, was not assessed and represent another study limitation.

## 5. Conclusions

The results of this study highlight the remarkable benefits of integrating physiotherapy with Negative Pressure Wound Therapy (NPWT) for patients with post-traumatic wounds. Our analysis demonstrated significant improvements in functional parameters, such as joint mobility and edema reduction, alongside a notable increase in patient-reported quality of life. The group treated with combined physiotherapy and NPWT experienced faster pain reduction and accelerated functional progression, promoting a more efficient and rapid recovery. These findings are consistent with previous studies that emphasized the synergistic effects of combined therapeutic approaches in wound management. Moreover, the results indicate a positive impact on psychological well-being, evidenced by reduced anxiety and depression levels in the group receiving physiotherapy. Such improvements reflect the value of a multidisciplinary approach, incorporating both physical interventions and psychological support to optimize the recovery process. These conclusions provide strong evidence for clinicians regarding the essential role of physiotherapy in complementing NPWT. They underscore the utility of a personalized treatment strategy to achieve superior outcomes in patient recovery.

## Figures and Tables

**Table 1:** Comparison of the study cohort background characteristics.

Variables	NPWT (n = 110)	NPWT + Physiotherapy (n = 95)	*p*-Value *
Age, years	36.59 ± 5.2	35.8 ± 3.7	0.126
Sex, men (%)	58 (52.7%)	63 (66.3%)	0.049
Overweight (>25.0 kg/m^2^)	51 (46.36%)	54 (56.84%)	0.134
Smoking	43 (39.1%)	40 (42.1%)	0.661
CCI > 2	2 (1.81%)	2 (2.1%)	0.632
Wound type			0.933
Open tibial fracture	41 (37.3%)	38 (40%)	
Post-surgical wounds	47 (42.7%)	39 (41.1%)	
Crush injuries	22 (20%)	18 (18.9%)	

* Chi-square or Fisher’s exact test, with a significance threshold of 0.006 after Bonferroni correction; CCI—Charlson Comorbidity Index.

**Table 2:** Ankle and knee mobility measurements at 10 days, 42 days, and 180 days between patients treated with NPWT exclusively and NPWT plus physiotherapy.

Variables	NPWT (n = 110)	NPWT + Physiotherapy (n = 95)	*p*-Value *
Dorsiflexion (°)—10 days	10.05 ± 1.76	14.22 ± 1.66	<0.001
Plantar flexion (°)—10 days	29.58 ± 2.42	35.38 ± 2.65	<0.001
Knee flexion (°)—10 days	115.57 ± 5.32	127.59 ± 5.31	<0.001
Pronation (°)—10 days	11.40 ± 2.16	16.09 ± 1.32	<0.001
Supination (°)—10 days	20.22 ± 2.56	25.45 ± 2.97	<0.001
Dorsiflexion (°)—42 days	15.12 ± 1.73	20.86 ± 1.23	<0.001
Plantar flexion (°)—42 days	35.56 ± 2.48	48.80 ± 2.20	<0.001
Knee flexion (°)—42 days	130.01 ± 4.49	137.91 ± 2.87	<0.001
Pronation (°)—42 days	16.61 ± 1.59	18.84 ± 0.96	<0.001
Supination (°)—42 days	27.90 ± 2.25	33.25 ± 2.61	<0.001
Dorsiflexion (°)—180 days	18.15 ± 1.86	20.86 ± 1.23	<0.001
Plantar flexion (°)—180 days	44.60 ± 2.73	48.80 ± 2.20	<0.001
Knee flexion (°)—180 days	135.59 ± 4.52	137.91 ± 2.87	<0.001
Pronation (°)—180 days	18.11 ± 1.63	18.84 ± 0.96	0.622
Supination (°)—180 days	33.42 ± 2.31	33.25 ± 2.61	0.626

* Student’s *t*-test, with a significance threshold of 0.016 after Bonferroni correction.

**Table 3:** Calf, edema circumference, and edema depth measurements at 10 days, 42 days, and 180 days between patients treated with NPWT exclusively and NPWT plus physiotherapy.

Variables	NPWT (n = 110)	NPWT + Physiotherapy (n = 95)	*p*-Value *
Calf circumference (cm)—10 days	37.48 ± 1.84	37.25 ± 1.76	0.361
Edema circumference (cm)—10 days	41.15 ± 2.39	40.58 ± 2.48	0.099
Edema depth (mm)—10 days	1.49 ± 0.8	1.41 ± 0.59	0.415
Calf circumference (cm)—42 days	36.28 ± 1.8	36.39 ± 1.65	0.651
Edema circumference (cm)—42 days	37.49 ± 1.84	37.25 ± 1.76	0.361
Edema depth (mm)—42 days	0.9 ± 0.042	0.63 ± 0.28	<0.001
Calf circumference (cm)—180 days	35.23 ± 1.79	36.07 ± 1.62	0.01
Edema circumference (cm)—180 days	36.29 ± 1.8	36.40 ± 1.65	0.651
Edema depth (mm)—180 days	0.53 ± 0.24	0.16 ± 0.12	<0.001

* Student’s *t*-test, with a significance threshold of 0.016 after Bonferroni correction.

**Table 4:** MMT tibialis anterior and triceps surae measurements at 10 days, 42 days, and 180 days between patients treated with NPWT exclusively and NPWT plus physiotherapy.

Variables	NPWT (n = 110)	NPWT + Physiotherapy (n = 95)	*p*-Value *
Initial MMT tibialis anterior	3.27 ± 0.67	3.09 ± 0.65	0.051
Initial MMT triceps surae	3.46 ± 0.67	3.31 ± 0.64	0.103
MMT tibialis anterior—10 days	3.60 ± 0.66	3.98 ± 0.67	<0.001
MMT triceps surae—10 days	3.88 ± 0.67	4.20 ± 0.61	<0.001
MMT tibialis anterior—42 days	4.14 ± 0.60	4.68 ± 0.47	<0.001
MMT triceps surae—42 days	4.41 ± 0.53	4.87 ± 0.33	<0.001
MMT tibialis anterior—180 days	4.83 ± 0.37	4.92 ± 0.27	0.052
MMT triceps surae—180 days	4.95 ± 0.23	5.00 ± 0.00	0.021

* Student’s *t*-test, with a significance threshold of 0.016 after Bonferroni correction.

**Table 5:** WHOQOL-BREF survey results.

Variables	NPWT (n = 110)	NPWT + Physiotherapy (n = 95)	*p*-Value *
Physical domain—10 days	27.17 ± 5.83	35.04 ± 6.46	<0.001
Physical domain—42 days	55.52 ± 7.44	67.12 ± 7.51	<0.001
Physical domain—180 days	85.12 ± 5.41	92.87 ± 5.54	<0.001
Psychological domain—10 days	45.04 ± 6.14	53.44 ± 6.39	<0.001
Psychological domain—42 days	64.98 ± 5.96	73.1 ± 6.27	<0.001
Psychological domain—180 days	85.74 ± 5.37	92.28 ± 5.84	<0.001
Social domain—10 days	49.93 ± 6.11	56.82 ± 5.52	<0.001
Social domain—42 days	65.84 ± 7.03	75.49 ± 7.1	<0.001
Social domain—180 days	91.61 ± 5.05	96.95 ± 4.09	<0.001
Mean score—10 days	59.89 ± 5.86	66.64 ± 6.24	<0.001
Mean score—42 days	72.47 ± 6.86	79.47 ± 7.37	<0.001
Mean score—180 days	93.40 ± 3.95	98.10 ± 2.67	<0.001

* Student’s *t*-test, with a significance threshold of 0.016 after Bonferroni correction.

**Table 6:** SF-36 survey results.

Variables	NPWT (n = 110)	NPWT + Physiotherapy (n = 95)	*p*-Value *
SF-36 score—10 days	40.54 ± 5.78	48.8 ± 6.26	<0.001
SF-36 score—42 days	61.44 ± 7.2	70.82 ± 7.36	<0.001
SF-36 score—180 days	85.60 ± 5.35	92.18 ± 5.32	<0.001

* Student’s *t*-test, with a significance threshold of 0.016 after Bonferroni correction.

**Table 7:** HADS survey results.

Variables	NPWT (n = 110)	NPWT + Physiotherapy (n = 95)	*p*-Value *
Anxiety score—10 days	13.14 ± 2.39	11.47 ± 2.39	<0.001
Anxiety score—42 days	7.93 ± 1.89	5.47 ± 1.91	<0.001
Anxiety score—180 days	4.44 ± 1.35	2.62 ± 1.53	<0.001
Depression score—10 days	10.87 ± 2.31	8.69 ± 2.21	<0.001
Depression score—42 days	6.95 ± 1.89	4.09 ± 1.82	<0.001
Depression score—180 days	3.74 ± 1.42	1.61 ± 1.32	<0.001

* Student’s *t*-test, with a significance threshold of 0.016 after Bonferroni correction.

**Table 8:** VAS—scale for pain survey results.

Variables	NPWT (n = 110)	NPWT + Physiotherapy (n = 95)	*p*-Value *
VAS score—10 days	6.7 ± 1.05	5.68 ± 1.13	<0.001
VAS score—42 days	3.31 ± 1.34	1.74 ± 1.25	<0.001
VAS score—180 days	0.05 ± 0.31	0 ± 0	0.110

* Student’s *t*-test, with a significance threshold of 0.016 after Bonferroni correction.

**Table 9:** Correlations over 10 days and 6 months post-intervention.

Time	Variable	Correlated Outcome	Coefficient (r) *	*p*-Value
10 Days	Dorsiflexion	SF-36	0.444	<0.001
10 Days	Dorsiflexion	WHOQOL-BREF	0.352	<0.001
10 Days	Dorsiflexion	HADS Anxiety Scores	−0.279	<0.001
10 Days	Dorsiflexion	VAS Scores	−0.319	<0.001
10 Days	Plantar flexion	SF-36	0.431	<0.001
10 Days	Plantar flexion	WHOQOL-BREF	0.411	<0.001
10 Days	Plantar flexion	HADS Anxiety Scores	−0.249	<0.001
10 Days	Plantar flexion	VAS Scores	−0.317	<0.001
10 Days	Knee flexion	WHOQOL-BREF	0.267	<0.001
42 Days	Dorsiflexion	SF-36	0.464	<0.001
42 Days	Dorsiflexion	WHOQOL-BREF	0.424	<0.001
42 Days	Dorsiflexion	HADS Anxiety Scores	−0.441	<0.001
42 Days	Dorsiflexion	VAS Scores	−0.457	<0.001
42 Days	Plantar flexion	SF-36	0.521	<0.001
42 Days	Plantar flexion	WHOQOL-BREF	0.337	<0.001
42 Days	Plantar flexion	HADS Anxiety Scores	−0.535	<0.001
42 Days	Plantar flexion	VAS Scores	−0.434	<0.001
42 Days	Knee flexion	WHOQOL-BREF	0.318	<0.001
180 Days	Dorsiflexion	SF-36	0.373	<0.001
180 Days	Dorsiflexion	WHOQOL-BREF	0.423	<0.001
180 Days	Dorsiflexion	HADS Anxiety Scores	−0.390	<0.001
180 Days	Dorsiflexion	VAS Scores	−0.124	<0.001
180 Days	Plantar flexion	SF-36	0.326	<0.001
180 Days	Plantar flexion	WHOQOL-BREF	0.349	<0.001
180 Days	Plantar flexion	HADS Anxiety Scores	−0.370	<0.001
180 Days	Plantar flexion	VAS Scores	−0.023	0.743
180 Days	Knee flexion	WHOQOL-BREF	0.216	0.002

* Pearson correlation.

## Data Availability

Data availability are subject to hospital approval.
